# A Pilot Study on the *e-Kayak* System: A Wireless DAQ Suited for Performance Analysis in Flatwater Sprint Kayaks

**DOI:** 10.3390/s20020542

**Published:** 2020-01-19

**Authors:** Vincenzo Bonaiuto, Giorgio Gatta, Cristian Romagnoli, Paolo Boatto, Nunzio Lanotte, Giuseppe Annino

**Affiliations:** 1 Sport Engineering Lab, Department of Industrial Engineering, University Rome Tor Vergata, 00133 Rome, Italy; cristian.romagnoli2@unibo.it (C.R.); g_annino@hotmail.com (G.A.); 2 Department for Life Quality Studies, University of Bologna, 47037 Rimini, Italy; giorgio.gatta@unibo.it; 3 APLAB, 00196 Roma, Italynunziolanotte@aplab.it (N.L.); 4 Department of Human Sciences and Promotion of the Quality of Life, San Raffaele Roma Open University, 00166 Rome, Italy; 5 Department of Systems Medicine, University Rome Tor Vergata, 00133 Rome, Italy

**Keywords:** sport, biomechanics, DAQ systems, paddling, flatwater sprint kayaking

## Abstract

Nowadays, in modern elite sport, the identification of the best training strategies which are useful in obtaining improvements during competitions requires an accurate measure of the physiologic and biomechanical parameters that affect performance. The goal of this pilot study was to investigate the capabilities of the *e-Kayak* system, a multichannel digital acquisition system specifically tailored for flatwater sprint kayaking application. *e-Kayak* allows the synchronous measure of all the parameters involved in kayak propulsion, both dynamic (including forces acting on the paddle and footrest) and kinematic (including stroke frequency, displacement, velocity, acceleration, roll, yaw, and pitch of the boat). After a detailed description of the system, we investigate its capability in supporting coaches to evaluate the performance of elite athletes’ trough-specific measurements. This approach allows for a better understanding of the paddler’s motion and the relevant effects on kayak behavior. The system allows the coach to carry out a wide study of kayak propulsion highlighting, and, at the same time, the occurrences of specific technical flaws in the paddling technique. In order to evaluate the correctness of the measurement results acquired in this pilot study, these results were compared with others which are available in the literature and which were obtained from subjects with similar characteristics.

## 1. Introduction

Nowadays, the use of a simple chronometer or of video analysis no longer represents a suitable system for the assessment of training or a race performance for elite athletes. Indeed, this approach does not allow for investigation of all the parameters involved in the assessment of performance, and it permits qualitative analysis at best. Conversely, a deep “knowledge of performance” by both coach and athlete (i.e., not only the value of the result itself but the way in which this has been obtained [1]) represents a key factor in improving the training procedures of this class of athletes. For this reason, it is possible to obtain better improvements in performance only when athletes and coaches are able to receive live and accurate feedback during training sessions, or an easily understandable full report after training, for deeper analysis.

In recent years, several electronic measurement systems specially designed for sport applications have been proposed in the literature or have been made available on the market. With this in mind, there are some “golden rules" that must be taken into account in order to design a suitable electronic system for the monitoring of a sport performance. Firstly, the system has to be accepted by both coach and athlete. Thus, the system has to be reliable and easy to use, and its output must be easy to understand and able to allow for a deep investigation into the quality of the performance. Furthermore, to be easily accepted by the athlete, the sensors applied on him or her or on the sports equipment must be light, unobtrusive, and not liable to influence in any way, physical or psychological, the performance itself [2,3,4,5].

In flatwater sprint kayaking, an effective paddling technique together with good physiological power and a smart race strategy plays a significant role in reaching peak performances among elite athletes. However, the particular features of such a paddling technique make the full comprehension of the way each force is proportionally involved in the boat’s propulsion difficult [6]. Kayaking is a cyclic sport where the forces that propel the boat can be identified in the whole muscular kinetic chain of the athlete [7,8,9,10]. During acceleration, the athlete needs to generate propulsive forces greater than the resistive forces of air and water acting on the boat; the applied forces have to maximize the velocity and the forward acceleration, while minimizing all the other unnecessary rotations and accelerations along the axes of the boat. Furthermore, in contrast to rowing, where the oar is constrained to the hull via an oarlock, in kayaking the force transferred by the athlete to the water through the paddle during the stroke is transmitted to the boat through the body of the paddler itself via the seat and footrest [11,12]. Hence, measuring the force on the paddle or the stroke rate alone is not enough for the coach to identify the best actions with which to improve the performances for this class of athletes. For this reason, even if the dynamic behavior of the blades in the water can be measured, the proportion with which each of these forces contributes to the boat’s motion is widely debated and, consequently, the study of its propulsion very complicated [12,13,14].

A useful key for good comprehension of the biomechanical parameters upon which the performance of the kayaker depends could be given by an accurate and simultaneous measure of the forces exerted by the upper body musculature on the paddle and those applied by the legs on the footrest together with the kinematics of the boat. This means that an in-depth study of dynamic and kinematic parameters of both athlete and boat could allow the coach to give practical tips which will refine the paddling technique and help to avoid specific technical flaws.

In recent years, several studies [10,11,12,13,14,15,16,17,18,19,20,21] have been published on force–time curve development performed by kayakers on ergometers (non-specific conditions), in lab measurements, and on boats (specific race conditions). These papers (Table 1) describe the use of particular digital acquisition (DAQ) systems, with sensors applied on the boat either on the boat paddle or on the footrest. The table provides a brief description of each system together with information about the presence of kinematic or dynamic sensors and the capability of each system to operate under standard training conditions (on water).

Specifically, in 2009 Mickael Begon et al. [7] presented a kinematic analysis of the anteroposterior forces applied to the footrest, the seat, and the paddle of a kayak by using a special ergometer instrumented with seven uniaxial force sensors and two goniometers. The ergometer was provided with a trolley sliding forward and backward along a static frame. The aim of this study was the assessment of the paddling performances, the analysis of the involved forces, and the coordination between the left and right sides. F. Nates et al. (2015) [20] presented a study in which, by using a special kayak ergometer (the Poitiers-B kayak ergometer), they installed six degree of freedom (DOF) force sensors for the measurement of the contact force between the athlete’s hands and the paddle shaft. This allowed for a tridimensional study of the forces applied on the paddle and improvement of the knowledge in both the drive and recovery phases.

In 2010, Sturm et al. [19] were involved in the design of a system consisting of force sensors positioned on both the paddle and footrest and wirelessly connected to a central unit via a Bluetooth radio link. The goal of this study was to present a measuring tool able to record, in a training session of flatwater kayaking, the strength on the paddle and footrest. The forces on the paddle were measured by means of four strain gauges connected in a Wheatstone full-bridge, while, for the measure of the forces on the footrest, force-sensitive resistor (FSR) sensors were employed. The measures obtained by the proposed system were compared to those achieved using a Dansprint kayak ergometer [22], showing a good correlation between the power (measured by the ergometer) and the force on the paddles (measured by sensors). 

In 2011, Gomes et al. [14] investigated the intracyclic velocity variation as well as the kayak’s motion using a triaxial accelerometer in K1, K2, and K4 boats. In the same year, Gomes et al. [6] presented the *FPaddle* system. This is a wireless system that allows the measurement of the paddle forces in a specific environment. Later, in 2015, the same authors presented an updated version of the *FPaddle* system (with only two strain gages) where the paddling force profile and the force–time curves were studied together with triaxial accelerometry [13].

Finally, Luo Niu et al. (2019) [21] proposed to evaluate kayak paddling performance by use of a custom-built paddle instrumented with a special optical fiber technology, namely, Fiber Bragg Grating (FBG) strain sensors [23]). Through this new technology system, it is possible to measure handgrip load and blade load distribution in on-water kayaking in real time. The proposed technology seems to be very promising for the measurements of forces on the paddle. Nevertheless, it seems to be obtrusive (it is still a cabled system) and it cannot be employed in standard training conditions. In fact, the presence of the cables does not allow for a specific assessment of the performance [3,19].

In kayaking, the main kinematic parameters which are useful in the investigation of performance include velocity, acceleration, intra-cyclic velocity, roll, and pitch of the boat. In addition, the dynamic parameters have to include stroke frequency, forces acting on the paddle and footrest, the symmetry between the forces on the right and left side for both the paddle and footrest, and the synchronization among these forces [12,13,14].

As previously shown, most of these systems cannot simultaneously measure the kinematic and dynamic parameters that affects the kayaker’s performance. In particular, as reported in Table 1, only three of them are able to obtain both measurements, and, among these, only one has been designed for on-water use [13].

In this context, the aim of this pilot study was to investigate the capabilities of a wireless multichannel portable DAQ (the *e-Kayak* system) in supporting coaches to evaluate the performance of elite athletes. The system is tested on a couple of athletes through specific measurements during on-water training and simulating specific race phases in a specific flatwater kayak environment. In particular, the measurement results presented in this paper are related to some different tests, i.e., a slow-pace test (100 m and 150 m for the female and male subjects, respectively), a 50 m fast-pace test for the female subjects only, and, for the male subjects, a 40 m speed test simulation starting from the race blocks. In the slow-pace tests, the subjects are asked by the coach to perform the test with care taken to perform the proper paddling technique (i.e., performing a specific work on the technical gesture). The tests are carried out in a lake with a negligible level of water currents, and the system synchronously acquires the force signal on both the paddle and footrest, the position and the boat’s speed by a Global Position System (GPS) device and three-axis acceleration, and the yaw, pitch, and roll by an inertial measurement unit (IMU) installed inside the boat.

The goal of the test was the evaluation of the type of parameters supplied by it in order to verify their usefulness for the measurement of the performance and the identification of technical flaws. Moreover, the test verifies the usability of the system as per the readability of the output by the coaches and easiness of the setup and calibration procedures.

## 2. Materials and Methods

### 2.1. Measurement System

The DAQ used for measurements here is the “*e-Kayak* system”, which was developed in the framework of a project carried out on behalf of the Italian Olympic Committee. We have employed a version customized for flatwater sprint kayaking of the DAQuino system [24,25], a modular architecture easily customizable for particular sport application that allows for the connection of up to eight slave nodes to a master one via a high performance 2.4 GHz wireless link (ISM Band). The design of this new system was inspired by previous experience gained using a first preliminary prototype developed in 2016 [26]. Although that prototype presented the same class of sensors (IMU, GPS, and force sensors) of the DAQuino system, the modular architecture, the maximum available sample rate of most of the new employed sensors, the unobtrusiveness in the position of the force sensors, and the new implemented communication protocol make the new system more reliable and effective. For example, in the previous prototype system, the slave node for the measurement of the force exerted on the paddle was installed on the outside in the middle of the shaft (a barycentric but rather obtrusive position) while the connection with the footrest was implemented by a wire. Furthermore, in a manner different from the previous system, the modular architecture of the new system easily allows its use on K2 and K4 boats.

**Figure 1:**
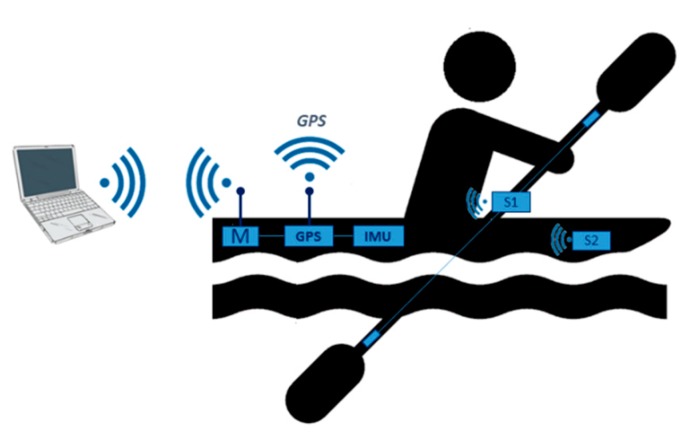
Block scheme of the *e-Kayak* system. Legend: GPS, Global Positioning System; IMU, inertial measurement unit; S1, paddle; S2, footrest.

In particular, the new system is equipped with a 9-axis IMU, a high sample rate GPS device, and a pair of force sensors applied on the paddle and footrest for each of the kayakers belonging to the crew of the boat. Hence, as depicted in Figure 1, the system designed for a K1 boat (i.e., a kayak with only one paddler) presented in this paper is composed of a master node (M) and two slave nodes equipped with force sensors and installed on the paddle (S1) and footrest (S2).

The master node handles the data stream from the dynamic sensors on the slave nodes and the acquisition of the kinematic data from the IMU and GPS. Moreover, it provides, via a wi-fi link over a dedicated WLAN (Wireless Local Area Network), a communication interface on the user terminal (i.e., a tablet, smartphone, or PC, etc.) for real-time feedback on a reduced set of parameters. The same wi-fi link can be used to download the data of the whole training or race session.

Since the interface is a webpage, it can be used with any operating system and any device, as long as a web browser is present. In addition, the master node manages the synchronization of the data acquisition; due to the large amount of computational resources needed to carry out all these tasks, the node has been designed to host a high-performance micro controller unit (MCU, 32 bit 180 MHz ARM Cortex-M4 Pjrc’s Teensy 3.6). The wi-fi transmission is based on the module ESP8266 (Espressif Systems Co., Ltd., Shanghai, China), while the communication with the slave nodes is granted by nRF24L01P+ modules (Nordic Semiconductor [27]). These modules are low power single chip transceivers in the global license-free 2.4 GHz ISM band with high-speed communications capability (up to 2 Mbit/s). They are suitable for deploying wireless networks in several application fields. In the *e-Kayak* system, they have been equipped with an external antenna that increases the transmission range of the device in order to overcome the signal losses due to the boat and the body of the athlete itself.

**Figure 2:**
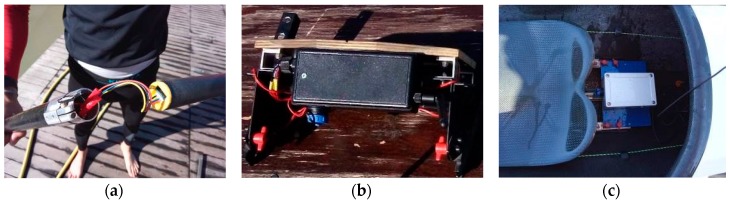
*e-Kayak* system: (**a**) paddle node, (**b**) footrest node, and (**c**) master node installed on the boat.

The GPS device which is installed in the system is based on the Venus822A chip by Skytraq Technology Inc. Taiwan. It has been designed for quad-GNSS (Global Navigation Satellite System) applications and can work with an up to 50 Hz update rate when it is running at top speed (this feature is valid if it processes signals from the US satellite system GPS only). In the described system, it is employed with an update frequency set at 20 Hz that provides an accurate measurement of the velocity fluctuations at each stroke (intracyclic velocity). Finally, the IMU is a nine DOF device that allows a sampling frequency of up to 50 Hz based on the MPU 9250 by TDK Co., Tokyo, Japan. LiPo batteries power the master as well as each of the slave nodes, allowing an operating time of more than 2 h. The user can monitor the battery charge level on the master and the slave nodes using the web interface. The master node is placed in a waterproof case for on board use (Figure 2c) and is installed inside the boat and fastened to the central rail on the back of the seat. Since its weight is limited (about 450 g), it can be compensated with an appropriate sizing of the ballast.

Both the paddle and footrest nodes are composed of a microcontroller unit (16 MHz ATmega328 processor, Atmel, San Jose, CA, USA), a radio circuit (again based on the module nRF24L01P+), and a signal conditioning circuit based on the instrumentation amplifier INA326 (Texas Instruments, Dallas, TX, USA) connected to the force sensors for signal amplification and noise filtering.

In particular, for the measure of the deformation of the shaft, these sensors (four waterproof strain gauges (KFW-5-120, Kyowa, Tokyo, Japan)) are connected in a Wheatstone full-bridge configuration and each pair of them is placed in the left and right side of the shaft at the same distance from the tip blades (i.e., at 53 cm). Each pair is placed parallel to the bending plane of the nearby blade. Of the two strain gauges composing a pair, one measures the tensile strain (+ε) and the other the compressive strain (−ε). The full bridge configuration ensures temperature and lead resistance compensation. The force applied on the blade can thus be measured by measuring the bending strain on the shaft.

**Figure 3:**
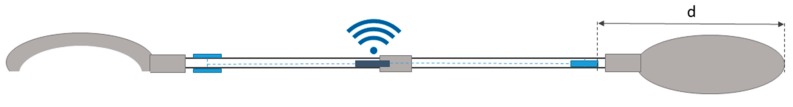
Block scheme of the paddle slave node (where *d* is the distance of the strain gauge sensor from the tip blade).

The paddle’s slave node has been designed to be installed inside the shaft of the paddle (Figure 2a). Its weight is only 30 g, and since it can be placed in a barycentric and unobtrusive position, it does not affect in any way the movements of the kayaker. Figure 3 depicts a scheme of the instrumented paddle. Conversely, for the measurement of the forces on the footrest, the same strain gauges used in the paddle are connected in a Wheatstone half-bridge configuration and are placed, in order to measure the strain caused by the forces applied by the legs, on the back of the footrest itself. Each slave node (paddle and footrest) is individually calibrated before each training session according to the procedure described by Aitken [8] and Nilsson [28] and accurately described by Gomes et al. in [13]. Such a calibration procedure for the paddle consists of statically loading it, in specific positions for both sides, with calibrated masses by finding the relationship between the force applied on the paddle shaft and the signal measured on the circuit. The footrest is calibrated with a similar procedure by loading it with calibrated masses. The setup of the system takes a few minutes to position the master node on the boat and to carry out the zero adjusting procedure of the IMU. The system, though for the kinematic part only, has been widely tested during a few high level competitions both in training as well as under race conditions. Although a deep and complete study on the usability of such a system has still not been carried out, this experience has resulted in a rather easy-to-use system, and both the setup and the calibration procedures also seem to be affordable for non-technician people.

**Figure 4:**
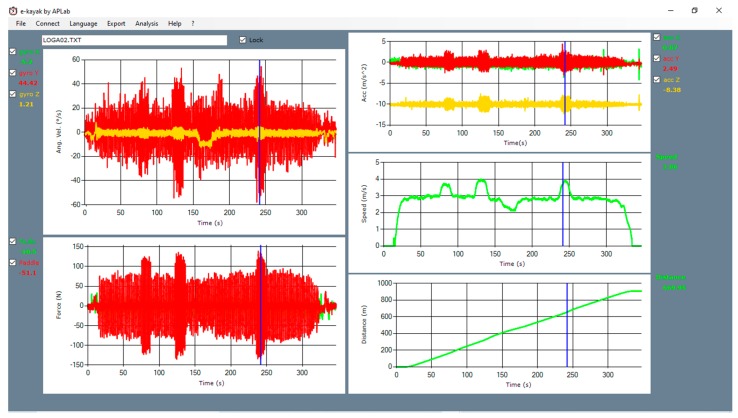
Screenshot of the *e-Kayak* data visualization software.

The acquisition software is simply a web page, and it allows for immediate monitoring by both athlete and coach of a restricted number of parameters, including instantaneous stroke rate, boat speed, and traveled distance. A more efficient visualization application (Figure 4) allows for analysis of all the dynamic and kinematic data acquired during the whole training session. The data of the angular accelerations are depicted in the top graph on the left side of the screen, while the forces on the paddle (red) and footrest (green) are shown in the bottom left corner. The three charts on the right side show, from top to bottom, the acceleration, the instantaneous velocity, and the distance travelled. A synchronized marker allows the user to highlight specific events of the session and show the measured value for each of the acquired parameters. The instrumented paddles were for both athletes BRACA-SPORT Mod. BRACA-IV; the kayak used for the female subject was NELO4, while for the male subject it was PLASTEX. 

### 2.2. Methods

The data were collected during training sessions of K1 boats in flat water. The force on both the paddle and footrest, as well as the kinematic data from the IMU, were synchronously acquired at a sampling frequency of 50 Hz while the velocity and the distance were sampled from the GPS at a frequency of 20 Hz. The data were acquired during on-water training sessions at different paces; two athletes (female, age 24, height 163 cm, weight 67 kg; male, age 28, height 185 cm, weight 85 kg) with international level flatwater kayak competition experience were involved in the sessions. The subjects, after a brief period of warmup and familiarization with the instrumentation, were asked to perform a standard training session featuring repetitions at different lengths and with increasing force intensity and stroke rate; the subjects also simulated some specific race phases (e.g., the starting phase). The athletes were asked to use their usual paddling technique in terms of stroke length and rate during the tests. Each subject performed three different repetitions, each followed by complete recovery; the best repetition for each athlete was taken into account in this study. Written informed consent was obtained after familiarization and explanation of the benefit and risks involved in the procedures adopted. The study was approved by the Ethical Committee of the School of Sports and Exercise Science at the University of Rome “Tor Vergata”, Faculty of Medicine and Surgery. Moreover, all the tests were carried out in accordance with the Declaration of Helsinki.

### 2.3. Data Analysis

The collected data were analyzed using the previously described visualization software, while some of the procedures for the data analysis were automated using a custom program (Matlab R2018b by The MathWorks Inc., Natick, MA, USA). The former provided the smoothing and filtering of the acquired data by a fourth-order low-pass Butterworth filter, while the latter was employed to automatically detect the strokes by analysis of the paddle force–time curves and for the computation of all the parameters (i.e., time values and forces). Each pulse of the force–time curve was identified as a force impulse when all the following features had been detected: the peak force was above a specific threshold (fixed at 70% of the maximum value of force) and the curve showed a fast rise followed by a falling to its resting value. The sum of the wet time and aerial time represents the stroke duration. The onset and the end of the force application were automatically detected by software routine. In particular, they were identified as the points where the force–time curve respectively leaves (paddle entry into the water) or returns (paddle exit) to its resting value. Moreover, two consecutive pulses in the force–time curve were identified as different strokes only if a time interval was measured (equal to the glide time) at which such a curve remained at the resting value.

## 3. Results

The *e-Kayak* system was installed on the boats of the two kayakers and some measurements results acquired during the tests are reported in this paper. In order to evaluate the capabilities of the system, parameters were identified among the parameters that the system is able to measure which, in the literature, are considered more affordable for the assessment of propulsion in kayaking. Among the parameters that can be measured for the assessment of performance in elite kayaking, the most significant ones are undoubtedly the force applied to the paddle by the kayaker as well as the stroke rate and good boat buoyancy and velocity [12]. In particular, with regard to the paddling analysis, several studies can be found in the literature that identify some specific parameters that can be used for an evaluation of the most effective technique. Among others, it is possible to identify the underwater phase duration (wet time (s)), the aerial phase duration (recovery time (s)), the time to force peak (time to peak (s)), the force impulse (i.e., the area under the force–time curve during the wet phase (N·s)) and the ratio between the average force and the peak one (F_average_/F_peak_ ratio) [12,13,29].

Table 2 and Table 3 give a summary of the biomechanical parameters measured from the tests at, respectively, slow and fast paces for the female subject while Table 4 and Table 5 show the same parameters measured for the male subject.

**Figure 5:**
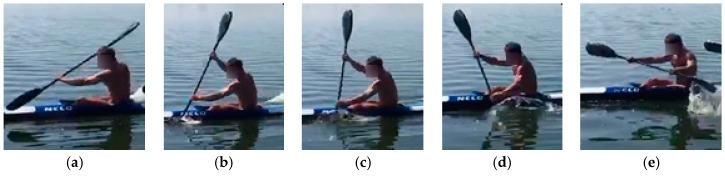
Kayak paddling stroke phases: (**a**) entry, (**b**) catch, (**c**) pull, (**d**) exit, (**e**) recovery.

**Figure 6:**
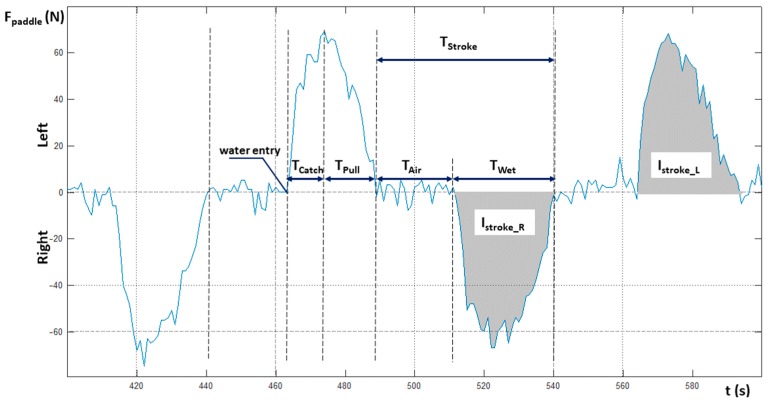
Kayak paddling stroke phases. Legend: T_catch_ [s] = length of the catch phase, T_pull_ [s] = length of the pull phase, T_air_ [s] = length of the air (recovery) phase, T_wet_ [s] = length of the wet phase (T_catch_ + T_pull_), T_stroke_ [s] = length of the stroke phase (T_air_ + T_wet_), I_stroke_R_ = right stroke pulse, I_stroke_L_ = left stroke pulse.

Figure 5 depicts these paddling phases, namely, the drive or underwater (wet) phase (Figure 5b–d) followed by an air or recovery phase (Figure 5a,e); in particular, in the wet phase it is possible to identify three different sub-phases, namely, the paddle entry into the water (Figure 5b), the catch (Figure 5c), and the pull (Figure 5d). Figure 6 depicts the aforementioned parameters and, via gray coloration, highlights the paddle force impulses of the right and left side, respectively. The force impulse is the area under the force–time curve.

## 4. Discussion

Table 2 and Table 3 report the measured biomechanical variables for the female subject paddling a K1 over different lengths (ranging 50 to 100 m) at slow and fast paces, respectively. It is worth observing that with an increase of about 32.25% for the stroke rate (SR) of the subject, the peak force increases by 30.36%, the force impulse (FI) by 22.43%, and the product FI-SR by more than 61.92%. Nevertheless, the resulting increase in the boat’s velocity is only about 2.45%. This is mainly due to a difference in the operating conditions that occurred between the two tests (i.e., changes in water current intensity). In any case, it is worth noting a reduction in the time of the paddle in the water (T_wet_), which it is possible to observe also by the decrease in the T_wet_/T_stroke_ ratio of about 12.37% (from 52.42% to 64.79%). Hence, the increase in the SR was obtained by performing a more significant decrease in the T_wet_ rather than the recovery time. This result could be justified by a higher imbalance in the values of the left/right roll that was measured during the fast pace paddling.

Table 4 and Table 5 report the same biomechanical variables for the male subject under similar training conditions (lengths ranging from 40 to 150 m). In this case, it is possible to observe that with an increase of 46% for the SR average increase were measured of 89% for the peak force, 56.66% for the FI, and 128% for the FI-SR product. At the same time, the boat’s velocity showed a significant increase of 27.7%. The fast pace data of Table 5 are relative to a test where the subject was simulating a race start phase, i.e., the first 40 m were performed at maximal intensity.

In stark contrast to the female subject, the increase in SR was obtained in this case with a more significant reduction of the recovery time with respect to T_wet_. Conversely, according to Baker [12], a measured value for the T_wet_/T_stroke_ ratio of above 75% represents a signal of a too fast transfer between the two strokes with negative effects on the paddling efficiency (it is also taken into account that this could lead to an early onset of fatigue).

**Figure 7:**
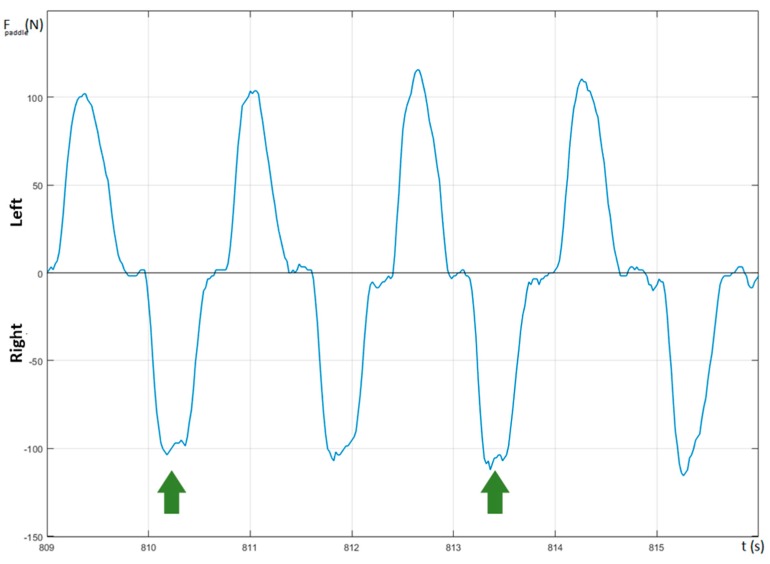
Bimodal (highlighted by arrows) and unimodal paddle force curves.

The shape of the paddle’s force–time curve provides significant information about the paddling technique of the kayaker. As reported in Baker [12], there are two possible shapes that can occur: curves that present a double peak (“*bimodal force curve*”) and curves with a single peak (“*unimodal force curve*”). The *bimodal force curve* is characterized by the presence of an initial small peak in the rise phase of the curve followed by a slight fall and a further rise to reach a new peak. Such a shape is an indicator of a possible technical flaw, i.e., a light flexion, immediately after the catch, of the elbow, which should be perfectly extended, or an early pushing of the top hand in the stroke. Both flaws are often the consequence of an over-aggressive catch. In fact, this could be justified by the elastic response of the shaft, which is particularly noticeable when the kayaker tries to reduce the recovery time and to accelerate the paddle to reach the water as fast as possible [13]. Figure 7 depicts a force curve relative to the female subject involved in a fast pace training session where, highlighted by the green arrows, it is possible to note a couple of occurrences of a *bimodal force curve*. Both of these occurrences are located in the right side of the force curve and could be correlated to the right-handed nature of the athlete. In each of them, the amplitude of the second peak is lower than that of the first one: this, according to what is suggested by Baker, could be the confirmation of the use of a shaft with a proper degree of stiffness.

The force impulse multiplied by the stroke rate is equivalent to the total work done by the kayaker in moving the boat. Hence, the higher this product the faster the boat. Consequently, the measure of only one of these two factors cannot give a correct assessment of the paddling performance (i.e., a high value for the impulse area with a low stroke rate will result in a slow boat). Generally, the higher the stroke frequency, the lower the impulse for the same applied force, as a consequence of the proportional decrease in wet time. 

The shape of an ideal force curve should be a bell shape because this allows, by regular and efficient application of force, for it to be transformed into a smooth acceleration of the boat. Moreover, as in this case the velocity waveform presents a sinusoidal shape, if the intracyclic velocity variation is low, this reduces the energy expended by the athlete for the propulsion [12,30]. Conversely, as described by Gomes in [13], a fast rise time for the stroke force, together with the same peak value of the paddling force, should result in a higher value for the force impulse and, consequently, a faster boat. Thus, in order to obtain the best effectiveness in paddling, both the catch time (known as the catch slip) and the exit time (known as the release slip) should be taken into account. In fact, these should be as fast as possible so that the applied force quickly reaches its maximum value, which has to be maintained for the whole pull time. In this case, starting from a bell, the force waveform should tend to assume a rectangular shape. For this purpose, it is useful to monitor the F_aver_/F_peak_ ratio parameter, which is widely used in rowing [31]. In this study, the values of this parameter increased with the increase in SR and the average velocity for both subjects. Moreover, the delay between the paddle entry in the water and the start of the kayak’s acceleration decreased. Finally, at the end of the wet phase, the delay between the start of the kayak deceleration and the end of the force application decreased with increasing SR. This result suggests that at low SR, keeping the paddle in the water at the end of the wet phase could slow down the kayak.

The measurement of the peak force value represents another significant parameter for the evaluation of the water resistance of the boat while the simultaneous study of both the acceleration and force–time waveforms is a helpful key for evaluating the paddling technique and highlighting the effectiveness of the pulling, together with any imbalance in the boat’s movement. It is also useful for assessing progress in eliminating any technical flaws. 

**Figure 8:**
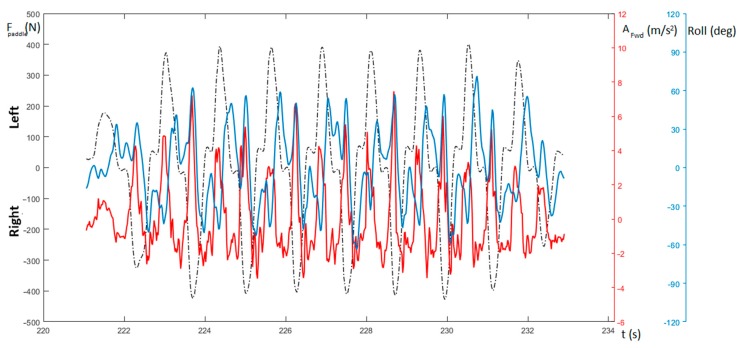
Measure of force on the paddle (F_paddle_—dashed), roll (light blue), and the boat’s acceleration (A_fwd_—red).

Figure 8 shows the measurement of forward acceleration (red curve) together with the measurement of the roll (light blue) and the force on the paddle (dashed). It is possible to notice that the peaks of the roll curve occur mostly in correspondence with the peaks of the paddle force and acceleration. It is worth noting that these rotations could also occur for particular body movements of the kayaker and that their intensity could not be directly connected to the amplitude of the forward acceleration.

The kayak velocity is mostly influenced by yaw and roll rather than pitch. In any case, even if it is clear that the rotations of the boat should be as small as possible, it is not easy to identify a paddling technique that represents the best trade-off between a great force applied on the paddle and a limited roll. This can only be obtained with a specific study of the athlete’s movements, and, for this purpose, the *e-Kayak* system seems to be a useful tool for such an analysis.

As depicted in Figure 8, the male subject showed a slight imbalance in the force pulled on the paddle. The forward acceleration curve (the red curve in the figure) confirms this left/right force imbalance with values of the acceleration peaks for the right side generally presenting a higher value with respect those of the left one. This disparity will cause a rotation of the boat around its vertical axis, thus increasing the resistance to the forward motion of the hull (i.e., affecting both the surface and wave drag), and, consequently, resulting in a deceleration [32].

**Figure 9:**
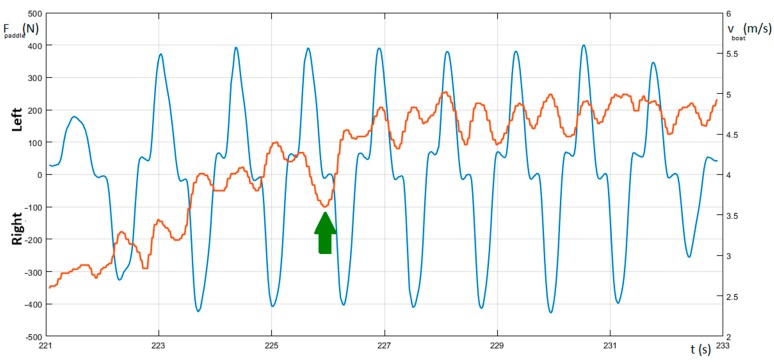
Measure of force on the paddle (F_paddle_—blue) and the boat’s velocity (v_boat_—red).

Figure 9 shows that for the male subject, the measurement of the velocity and paddling force of the first strokes occurs immediately after the start. The mean value of the velocity increases until it reaches the final value. The velocity of the kayak presents a fluctuant wave shape because it will tend, inside each stroke cycle, to increase when the paddle force applied by the kayaker exceeds the drag force, and, successively, to fall when, during the air phase, no paddle force is being applied (intracyclic velocity). The peak velocity inside each stroke cycle is reached before the blade exits from the water. In particular, as suggested by Kendal [30], it is possible to see that the velocity increases during the pull phase of the stroke and that it decreases before the exit of the blade from the water. This could point to the presence of a period near the end of the traction phase where the produced force is not enough to maintain the speed of the kayak. The minimum value of the velocity occurs near the re-entry of the blade in the water. Furthermore, it is possible to note a difference between the value of the reduction in the velocity for the right and left sides, respectively. This asymmetry could be caused by the different coordination and strength between the dominant and non-dominant parts of the body. Furthermore, the velocity peaks are concurrent with the force peaks, whereas the minimum values of the speed are recorded in the phase just before the paddle entry. The amplitude of the ripple for the velocity waveform represents an optimal indicator of the boat’s run: the lower such a value the better the efficiency of the paddling, because, as reported above, high variations in the velocity are an indication of inefficient energy handling [14]. Again, in Figure 9, it is possible to notice that at time 226 s and when highlighted by the green arrow, there is a significant fluctuation in the velocity. This is probably due to a movement forward and then backward of the body of the athlete. A velocity variation of such a shape is generally present at the start of the race because to make the kayak accelerate the athlete tends to move the body forward. As a matter of fact, because later the body mass must be moved back during the race, this body movement will cause a following deceleration of the boat.

Since the introduction of the wing paddle, paddling techniques have widely changed, and involve to a much greater degree the musculature of the body trunk and the legs in force production. In fact, because the force exerted by the kayaker with the paddle acts far from the axis of the craft, every stroke will create a momentum which tends to turn the kayak [30]. Although this is somewhat compensated for by the particular design of the hull, it also has to be balanced by a force exerted by the leg on the footrest on the active side. Moreover, the same force can give useful aid to the kayaker in stabilizing the torso rotations that occur during each stroke movement. The exact timing between these movements which can be used to improve the effects of the paddling force is still debated among coaches. In any case, it has been shown in the literature that it is important to investigate the synchronization and the timing of movements between the upper and lower body of the athlete in order to maximize performance.

**Figure 10:**
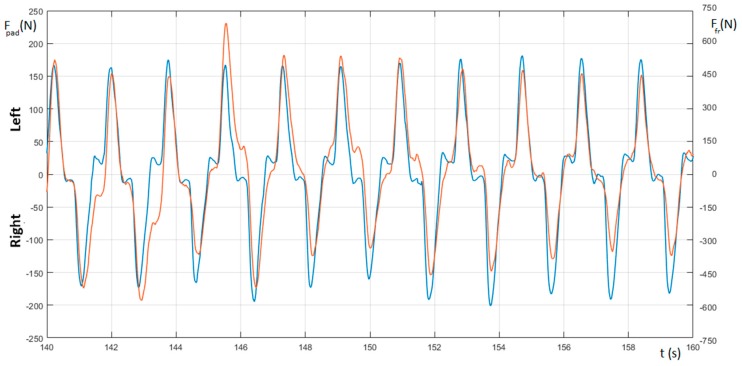
Measure of force on the paddle (F_pad_—orange) and the footrest ((F_fr_—blue).

**Figure 11:**
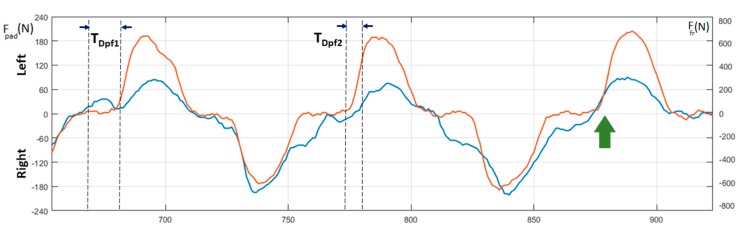
Detail of the force on the paddle (orange) and footrest (blue). Legend: F_pad_ [N] = force impressed on the paddle, F_fr_ [N] = force impressed on the footrest, T_Dpf1_ = leg-arm anticipation time [s], T_Dpf2_ = leg-arm delay time.

Figure 10 shows the force–time curves of the paddle (orange curve) and footrest (blue curve) by which it is possible to study the anticipation or the delay of each foot pressure with respect to the paddle entry in the water. Figure 11 depicts a detail of the same curves, where it is possible to focus on the level of coordination between the legs and arms during each stroke and measure the relative anticipation (T_Dpf1_) or delay (T_Dpf2_). In the same figure, highlighted by the green arrow, it is possible to note an example of the force exerted on the footrest that has been applied by the kayaker just before the beginning of the stroke.

No foot straps were used to maintain the position of the feet on the footrest, but we preferred to use the original footrest configuration as usually employed by the subjects in races or training. For this reason, as the shape of deformation detected by the strain gauges strongly depends on the position of force applied on the footrest, the amount of the measured force depends not only on the intensity of the pressure but also on the position of each foot with respect to the position of the force sensor. As a consequence, this force–time curve could not produce reliable values. In addition, it is worth noting that this position could not be the same for the length of the whole test. Consequently, these curves have to be taken into account for the timing of the gesture only, while the intensity of the measured forces as well as their symmetry between the two legs cannot be seen to reflect a real occurrence. On the other hand, though this could represent a limitation for the *e-Kayak* system, it is worth noting that this force is required, as discussed above, only for balancing the rotation of the boat. For this reason, the synchronization with the paddling force represents a parameter that can better provide a useful performance indicator with respect to that provided by the level of applied force. There are only a few studies available in the literature, [17,33] and all of them evaluate the forces on the footrest’s ergometers.

## 5. Conclusions

In flatwater sprint kayaking, paddler performances can be improved by a more effective paddling technique that consists of improvement in the propulsive power together with a reduction in the water drag by a proper checking of yaw and roll. This paper has presented a pilot study on the *e-Kayak* system, a multichannel DAQ system suited for flatwater sprint kayaking. The system manages the synchronous acquisition of both dynamic and kinematic parameters, allowing for an accurate assessment of paddling technique in real–life environments. The collected data allow a deep investigation into the assessment of paddling and can give useful evidence to differentiate between the skill levels of the athletes, while also allowing live feedback during training.

Because in this specific field an acknowledged reference system is not still available, in order to evaluate the reliability of the system, the measurement results achieved in this pilot study were compared with those available in the literature which were obtained by a system with similar features [13] for the same class of athletes. The performed comparison showed a good correspondence, for each stroke and for each of the cases depicted in this study, in the mean values of the force with respect to the exerted velocity, while the values of the other parameters were in a similar range in both studies. This suggests that the *e-Kayak* system can provide data of great interest to improving the knowledge of propulsion phases, namely, the wet and the recovery phases, during paddling. The possibility of highlighting, in fast time, several technical flaws, as for example those that are due to a lack of synchronization between arms and legs or those consequences of an over-aggressive catch, can support coaches to provide more effective suggestions to athletes in order to improve the paddling technique or in the identification of more suitable equipment. Moreover, the system seems to be highly promising for the development of objective criteria for the selection of crew members in team boats (K2 or K4 boats).

## Figures and Tables

**Table 1:** State of the art on DAQ systems developed for kayaking Legend: Dyn, dynamic; Kin, kinematic.

Authors	Brief Description	Dyn	Kin	On Water
Vos, J.A. et al. (1974) [7]	Strain gauges installed on both the paddle and footrest. A telemetry system allowed data transmission to a computer located at the shore. The study, initially formulated for rowing, has been applied to kayaking.	Yes	No	Yes
Campagna, P.D. et al. (1986) [15]	One of the first examples of an instrumented ergometer for kayaking. It has been demonstrated that this system is able to replicate open water paddling action very closely.	No	Yes	No
Aitken et al. (1992) [8]	Four strain gauges are attached near each blade for the measurement of the shaft bending. A data recorder system provides memorization of the acquired data for an offline download.	Yes	No	Yes
Pelham et al. (1993) [16]	Accelerometry measurement system based on electromagnetic force–balance accelerometers.	No	Yes	Yes
Begon, M., et al. (2009) [17]	Instrumented kayak ergometer for the measurement of the contact forces between the athlete and the ergometer.	Yes	Yes	No
Limonta, E. et al. (2010) [18]	Kayak simulator based on an automatic motion analysis system. It performs a three-dimensional kinematic analysis of the paddler’s movements.	No	Yes	No
Sturm, D. et al. (2010) [19]	A wireless (Bluetooth)-based sensor system is able to perform the measurement of the paddle bending by using four strain gauges. The measurement of the pressure of the foot on a custom-built footrest is obtained by force-sensitive resistor (FSR) sensors.	Yes	No	Yes
Gomes, B. et al. (2011) [6]	The *FPaddle* system measures the strain on the shaft via two couples of strain gages, with one placed on the bending plane of the blade and the other on a perpendicular plane. A wireless system provides the data transmission.	Yes	No	Yes
Gomes, B. et al. (2015) [13]	An *FPaddle* system instrumented with two strain gauges is integrated with a triaxial accelerometer placed inside the boat.	Yes	Yes	Yes
Nates, F.M. et al. (2015) [20]	A special kayak ergometer is designed with six degree of freedom (DOF) force sensors for the measurement of the contact force between the athlete’s hands and the paddle shaft.	Yes	No	No
Luo Niu et al. (2019) [21]	The measurement of the force on the shaft is obtained by the use of Fiber Bragg Grating (FBG) optical fiber sensors enclosed in the material of the blades.	Yes	Yes	No

**Table 2:** Female 100 m—slow pace (stroke rate (SR) = 62 str/min; velocity = 3.67 ± 0.26 m/s).

Biomechanical Variables	Left Paddle	Right Paddle
Force peak (N)	108.40 ± 5.04	106.46 ± 6.36
F_average_ (N)	77.19 ± 3.03	77.86 ± 5.44
F_average_/F_peak_ ratio (%)	71.43 ± 2.37	73.15 ± 3.21
Force impulse (N·s)	38.12 ± 2.24	38.75 ± 4.56
Time to peak (ms)	0.18 ± 0.03	0.17 ± 0.03
Stroke time (ms)	0.99 ± 0.07	0.99 ± 0.11
Wet time (ms)	0.51 ± 0.02	0.51 ± 0.05
Recovery time (ms)	0.47 ± 0.07	0.47 ± 0.08
T_Wet_/T_Stroke_ ratio (%)	52.55 ± 4.30	52.29 ± 4.42

**Table 3:** Female 50 m—fast pace (SR = 82 str/min; velocity = 3.76 ± 0.10 m/s).

Biomechanical Variables	Left Paddle	Right Paddle
Force peak (N)	142.25 ± 10.56	137.28 ± 9.93
F_average_ (N)	99.22 ± 7.36	103.09 ± 7.94
F_average_/F_peak_ ratio (%)	69.81 ± 2.80	75.12 ± 2.84
Force impulse (N·s)	46.20 ± 4.17	47.76 ± 6.37
Time to peak (ms)	0.21 ± 0.03	0.18 ± 0.02
Stroke time (ms)	0.74 ± 0.04	0.74 ± 0.04
Wet time (ms)	0.49 ± 0.05	0.48 ± 0.03
Recovery time (ms)	0.25 ± 0.05	0.27 ± 0.02
T_Wet_/T_Stroke_ ratio (%)	65.71 ± 5.55	63.87 ± 1.62

**Table 4:** Male 150 m—low pace (SR = 63 str/min; velocity = 3.24 ± 0.08 m/s).

Biomechanical Variables	Left Paddle	Right Paddle
Force peak (N)	166.39 ± 17.67	156.98 ± 13.58
F_average_ (N)	114.73 ± 9.46	111.86 ± 8.32
F_average_/F_peak_ ratio (%)	69.14 ± 2.53	71.35 ± 2.14
Force impulse (N·s)	48.68 ± 6.69	52.74 ± 5.16
Time to peak (ms)	0.16 ± 0.02	0.16 ± 0.01
Stroke time (ms)	0.97 ± 0.19	0.98 ± 0.04
Wet time (ms)	0.44 ± 0.04	0.49 ± 0.02
Recovery time (ms)	0.52 ± 0.15	0.49 ± 0.03
T_Wet_/T_Stroke_ ratio (%)	46.36 ± 3.96	49.83 ± 2.00

**Table 5:** Male 40 m—fast pace (SR = 90 str/min; velocity = 4.14 ± 0.25 m/s).

Biomechanical Variables	Left Paddle	Right Paddle
Force peak (N)	310.94 ± 13.16	301.13 ± 23.06
F_average_ (N)	217.57± 10.53	221.69 ± 14.01
F_average_/F_peak_ ratio (%)	69.97 ± 1.66	73.74 ± 2.75
Force impulse (N·s)	76.06 ± 5.97	82.74 ± 6.54
Time to peak (ms)	0.14 ± 0.01	0.15 ± 0.02
Stroke time (ms)	0.66 ± 0.03	0.67 ± 0.02
Wet time (ms)	0.37 ± 0.03	0.39 ± 0.04
Recovery time (ms)	0.29 ± 0.01	0.28 ± 0.01
T_Wet_/T_Stroke_ ratio (%)	56.45 ± 1.79	56.96 ± 2.54
